# Comparative Study Between ViewMind Atlas™, a Novel Digital Measure of Cognition, and Traditional Neuropsychological Tests

**DOI:** 10.1002/alz70856_102299

**Published:** 2025-12-25

**Authors:** María Bárbara Eizaguirre, Mario Parra, Natalia Ciufia, Aldana Lucia Marinangeli, Lucia Bacigalupe, Mariana Lucia Zarza, Gloria Lucia Mastroberti, Maria Fernanda Gallo, Lucia Ibarra, Lucas N Lapalma, Danilo Verge, Matias Shulz, Valentin Barco, Ricardo N Alonso, Gerardo Fernandez

**Affiliations:** ^1^ Ramos Mejía Hospital, Buenos Aires, Buenos Aires, Argentina; ^2^ ViewMind, NY, NY, USA

## Abstract

**Background:**

Cognitive impairment (CI) presents significant challenges in neurodegenerative conditions and aging populations. Traditional cognitive assessment tools often face limitations such as high costs, accessibility barriers, and lengthy administration times. ViewMind Atlas™, a digital biomarker combining eye‐tracking technology and artificial intelligence, provides a novel, efficient approach to cognitive evaluation. This study aimed to evaluate the capacity of ViewMind Atlas™ to detect CI, correlate its results with the Montreal Cognitive Assessment (MoCA) and other neuropsychological tests, and assess its clinical validity.

**Method:**

This cross‐sectional, single‐centre study included 154 participants aged 45–95 years with and without cognitive complaints. Cognitive assessments were performed using ViewMind Atlas™, which incorporates a head‐mounted display (HMD) with integrated eye‐tracking sensors. The system measured saccade amplitude, fixation duration, refixation rates, and response times during five validated visual tasks (Moving Dot, Go/No‐Go, New Colors, Color Combinations, and n‐back). Participants also completed the MoCA and a neuropsychological battery, including the Word Accentuation Test‐Buenos Aires (WAT‐BA), BEM‐144 Logical Memory and Serial Learning, Rey Complex Figure Test, Digit Span and Matrix Reasoning (WAIS‐III), Trail Making Tests A and B, Boston Naming Test, Phonological and Semantic Verbal Fluency Tests, and Clock Drawing Test. Statistical analyses included sensitivity, specificity, accuracy, and receiver operating characteristic (ROC) curves, as well as correlations between ViewMind metrics and cognitive test scores.

**Result:**

ViewMind Atlas™ demonstrated a sensitivity of 78%, specificity of 70%, and balanced accuracy of 74% for CI detection. Significant correlations were observed between ViewMind metrics and MoCA scores and other neuropsychological test results (*p* <0.05), validating its ability to identify CI.

**Conclusion:**

ViewMind Atlas™, leveraging HMD‐based eye‐tracking technology, is a valid, efficient tool for detecting cognitive impairment. Its strong alignment with traditional cognitive assessments supports its potential for clinical and research applications.

